# Bioluminescent monitoring of recombinant lactic acid bacteria and their products

**DOI:** 10.1128/mbio.01197-23

**Published:** 2023-09-05

**Authors:** In Young Choi, Jee-Hwan Oh, Zhiying Wang, Jan-Peter van Pijkeren

**Affiliations:** 1 Department of Food Science, University of Wisconsin-Madison, Madison, Wisconsin, USA; The University of Queensland, Brisbane, Queensland, Australia

**Keywords:** bioluminescence, lactic acid bacteria, probiotics, microbial therapeutics

## Abstract

Biotherapeutic strategies to promote health, including the application of engineered microbes to deliver therapeutic molecules, hold strong promise. However, without precision tools to detect therapeutic microbes and their products, we are hampered in our ability to monitor and fine-tune therapeutic delivery. Here, we adapted a bioluminescent peptide tagging system for use in lactic acid bacteria, a group of organisms whose members are commonly exploited as delivery vehicles of therapeutics and vaccines. As a proof of concept, we developed various *Limosilactobacillus reuteri* strains that each produced a recombinant therapeutic protein with an 11 amino acid tag, which is essential to yield a luminescent signal. Luminescent-based quantification of recombinant protein was more sensitive than commercially available immunoassays. In addition, we demonstrated that the bioluminescent peptide tagging system allows *in situ* recombinant protein detection in a continuous-culture parallel bioreactor system. This presents an exciting opportunity to determine recombinant protein production dynamics in response to different stimuli. Finally, following oral administration of recombinant microbes, luminescence in intestinal and fecal samples allowed for rapid detection of microbes with equal sensitivity to conventional plate count. Because we demonstrated the functionality of this bioluminescent peptide tagging system in 12 species encompassing nine genera*,* our approach will create previously unexplored opportunities in lactic acid bacteria research.

**IMPORTANCE:**

Lactic acid bacteria constitute a genetically diverse group of microorganisms with significant roles in the food industry, biotechnology, agriculture, and medicine. A core understanding of bacterial physiology in diverse environments is crucial to select and develop bacteria for industrial and medical applications. However, there is a lack of versatile tools to track (recombinant) protein production in lactic acid bacteria. In this study, we adapted a peptide-based bioluminescent tagging system that is functional across multiple genera and species. This system enables tracking of tagged proteins both *in vitro* and *in situ*, while it also can be used to enumerate recombinant bacteria from the mouse gastrointestinal tract with accuracy comparable to that of conventional plate counts. Our work expands the lactic acid bacteria genetic toolbox and will facilitate researchers in industry and academia with opportunities to monitor microbes and proteins under different physiologically relevant conditions.

## INTRODUCTION

Lactic acid bacteria (LAB) constitute a large family of gram-positive bacteria and nonspore or endospore-forming bacteria. Apart from some notable exceptions, many LAB are considered nonpathogenic and are naturally occurring in diverse habitats, including the gastrointestinal tract (GIT) of humans and other vertebrates (1, 2), as well as environments such as milk, soil, and fermented foods (3). Due to their long history of safe consumption, select strains of LAB are approved as Generally Recognized as Safe by the U.S. Food and Drug Administration (4) and are widely utilized as starter cultures and probiotics (3). Beyond their probiotic applications, LAB show promise as delivery systems of therapeutic proteins (5 – 7). To better understand the dynamics by which microbes transition and produce recombinant proteins *in vitro* and throughout the GIT, tools to track LAB and their products are essential.

Fluorescent methods, such as green fluorescent protein (GFP) or mCherry, have been employed to track recombinant LAB in the GIT (8 – 11). Although fluorescent protein labeling has proven effective to track bacteria, this approach has limitations when quantifying recombinant proteins. Indeed, the fusion of native proteins with fluorescent proteins may lead to mislocalization, aggregation, misfolding, or loss of protein function (12). In addition, phototoxicity from fluorescent tagging may negatively impact live host cells and tissues (13) and the fluorescent assay often faces challenges related to reduced sensitivity, reduced solubility, and elevated background levels compared to luminescent assays (14).

To track recombinant proteins, bioluminescence-based assays have proven fruitful, including the HiBiT system, which was originally developed for use in eukaryotic cells. The HiBiT system employs an 11 amino acid tag, which generates a luminescent signal upon high-affinity complementation with LgBiT, an 18 kDa subunit (15). Compared to larger protein fusions, the small tag is less likely to interfere with protein folding or functionality (16). In addition, the HiBiT system has a relatively low detection limit (femtomolar) (17), a broad dynamic range (18), and a rapid detection time (< 15 min). More recently, the HiBiT system has proven to be functional in some gram-negative bacteria (19, 20) and gram-positive bacteria (21, 22).

Here, we developed the bioluminescent peptide (HiBiT) tagging system for *in vitro*, *in vivo*, and *in situ* applications in multiple LAB species. Key features described in this study are the broad applicability and ability of the bioluminescent peptide tagging system to detect recombinant proteins *in situ* without cell lysis. In addition, bacteria isolated from culture or intestinal tissues can be quantified in minutes with an accuracy comparable to the conventional plate count method. The bioluminescent peptide tagging system showed superior sensitivity to detect recombinant proteins compared to commercial enzyme-linked immunosorbent assays (ELISAs). Because of the robustness, we envision that the bioluminescent peptide tagging system described in this study will be much welcomed by the LAB communities in academia and industry, and by the microbiome community in general.

## RESULTS

### Establishing bioluminescent peptide tagging in *Limosilactobacillus reuteri*

**Fig 1:**
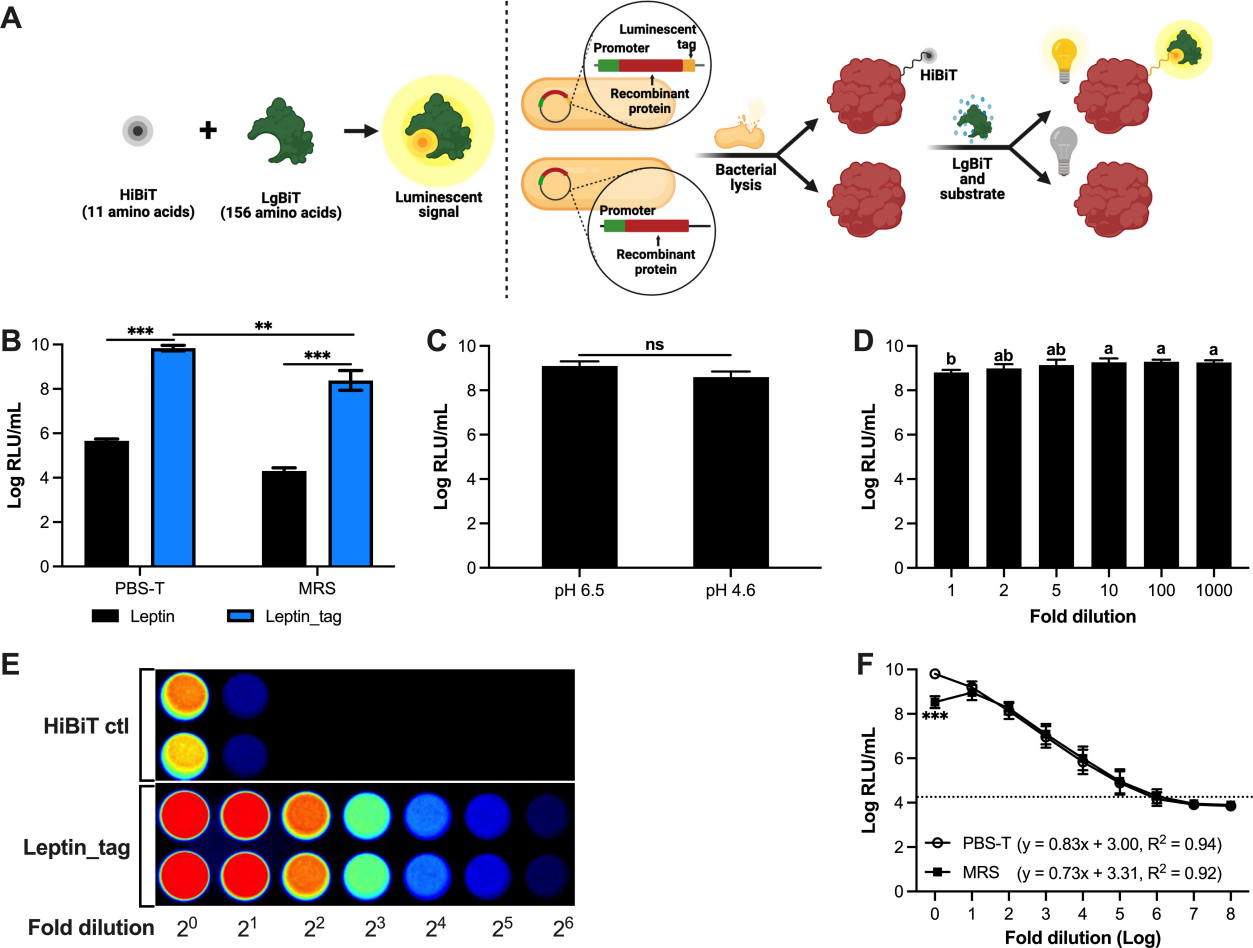
Development of a bioluminescent peptide tagging system in *Lm. reuteri*. (**A**) Schematic diagram of the bioluminescent peptide tagging system and (**B**) Comparison of luminescent signal derived from *Lm. reuteri* harboring pJP_Leptin or pJP_Leptin_tag. (**C**) Effect of pH on the luminescent signal. (**D**) Effect of MRS on the luminescent signal. (**E**) Visualizing luminescence of the HiBiT control protein and of lysates derived from *Lm. reuteri* harboring pJP_Leptin_tag. (**F**) Dynamic range of luminescence emitted by leptin_HiBiT in MRS and PBS-T. The dashed black line marks the background luminescence level. The correlation equation between bacterial concentrations and luminescent signals was determined by a simple linear regression analysis. Data are presented as means ± SD based on three biological replicates. **P < 0.01, ***P < 0.001, ns; no significant differences (*P* > 0.05) (two-tailed paired *t*-test). The letters (a and b) represent statistical differences among the groups (*P* < 0.05, one-way ANOVA).

The overall goal of this study was to adapt a bioluminescent peptide tagging system for broad application in LAB (Fig. 1A). As a first step, we cloned the sequence encoding the 11 amino acid tag (VSGWRLFKKIS) directly upstream of the stop codon of murine leptin, which is encoded from plasmid pJP_Leptin (2), to yield pJP_Leptin_tag. Next, cultures of *Limosilactobacillus reuteri* harboring pJP_Leptin or pJP_Leptin_tag, or their respective cell pellets resuspended in phosphate-buffered saline with Tween 20 (PBS-T), were disrupted by mechanical lysis. Lysates derived from the cultures and cell pellets derived from *Lm. reuteri* harboring pJP_Leptin_tag both yielded a 4-log increase in luminescence, compared to lysates derived from the untagged control (*P* < 0.001; Fig. 1B). Total luminescence level of cell culture in deMan Rogosa Sharpe (MRS) medium was approximately 10-fold lower compared to the respective samples that were resuspended in PBS-T. Overall, these data demonstrated the proof of concept of the application of bioluminescent peptide tagging in *Lm. reuteri* and established that cell suspensions in PBS-T yielded a slightly more robust signal compared to cell suspensions in MRS.

### Robust dynamic range of luminescence in MRS and PBS-T

Because the culture pH of *Lm. reuteri* decreased from 6.5 to 4.5 after 8-h incubation, we investigated the effect of pH on luminescence detection (Fig. 1C). The cell pellet from 8-h culture [9-log colony forming unit (CFU)/mL] was resuspended in fresh MRS (pH 6.5) or pH-adjusted MRS (pH 4.5). Luminescent readings revealed that luminescence levels obtained from MRS pH 6.5 were comparable to those obtained at pH 4.5 (*P* > 0.05). Thus, luminescence is not affected in MRS within the pH range of 4.5–6.5.

To test whether the dark media color, or select ingredients in MRS, interfered with luminescent readings, we resuspended nine 9-log CFU of *Lm. reuteri* harboring pJP_Leptin_tag in 1 mL of MRS or MRS that was diluted up to 1,000-fold, followed by cell disruption and luminescent detection (Fig. 1D). Compared to the undiluted sample, we identified that at least a 2-fold dilution increased luminescence levels by 1.6-fold (*P* < 0.05). Compared to the 2-fold diluted MRS suspension, additional dilutions did not significantly change luminescence levels. Thus, the color of MRS, perhaps combined with inhibitory substance(s) present in MRS, can be recognized as interference factors for luminescence detection, which can be alleviated by diluting the sample 2-fold.

As an initial step to gain insight into the dynamic range of luminescence, we used a bioluminescent imaging system to visualize twofold dilutions of lysates derived from engineered *Lm. reuteri* harboring pJP_Leptin_tag (Fig. 1E). Over the full dilution range (2^0^–2^6^), we observed a linear correlation between the dilution and the bioluminescence levels (*R^2^* = 0.98). To assess a broader range, we resuspended cell pellets of *Lm. reuteri* harboring pJP_Leptin_tag (9-log CFU/mL) in MRS or PBS-T, followed by 10-fold serial dilution in PBS-T and mechanical cell disruption (Fig. 1F). A linear correlation between bacterial cell numbers and luminescence was identified for PBS-T in the dilution range from 10^0^ to 10^6^ (*R^2^ =* 0.94), while for MRS the dynamic range spanned from 10^1^ to 10^6^ (*R^2^* = 0.92).

### Quantification by bioluminescent peptide tagging is more robust than ELISA

**Fig 2:**
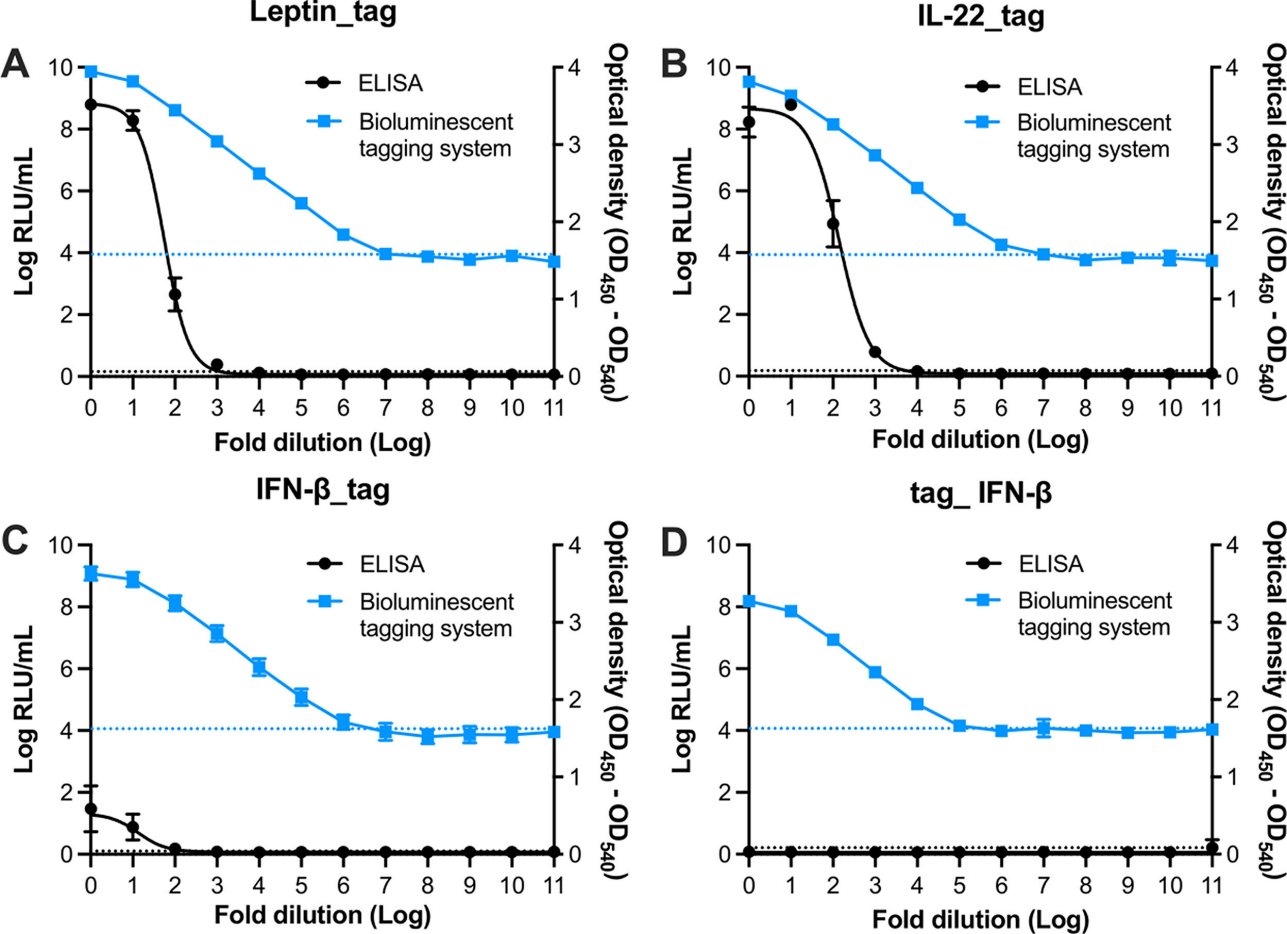
Comparison of dynamic range between a luminescent assay and ELISA. (A–D) Dynamic range of (**A**) leptin_tag, (**B**) IL-22_tag, (**C**) IFN-β_tag, and (**D**) tag_ IFN-β represented in relative light unit (RLU) and OD. Data are presented as means ± SD based on three biological replicates.

ELISAs are routinely used to quantify (recombinant) proteins, including cytokines. In addition to *Lm. reuteri*-producing murine leptin, we previously engineered *Lm. reuteri* to produce murine interleukin-22 (IL-22) (6). In this work, we engineered *Lm. reuteri* to produce murine interferon- (IFN-β). We additionally developed *Lm. reuteri* harboring pJP_IL-22_tag and *Lm. reuteri* harboring pJP_IFN-β_tag. To compare the performance between commercial ELISAs and bioluminescent peptide tagging, lysates derived from each recombinant *Lm. reuteri* were diluted and subjected to each assay. Although the optical density (OD) of the ELISA correlated with the dilution of the recombinant proteins (Fig. 2A through C ), the dynamic range of ELISA was limited to the nanogram level, whereas the luminescent assay detected recombinant proteins down to the femtogram level (Table 1; Fig. S1). We found that the total protein levels determined by ELISA and luminescent assay were comparable for leptin_tag and IL-22_tag. However, the IFN-β_tag levels detected by ELISA were lower than that from the luminescent assay (*P* < 0.05). We also found that the presence of the 3′-tag on IFN-β interfered with the immune assay, as the level of IFN-β detected by ELISA was two times higher than IFN-β_tag. To test whether moving the tag would overcome this issue, we generated a derivative that had the tag at the 5′ end of IFN-β. While the luminescent level of 5′-tagged IFN-β was comparable to 3′-tagged IFN-β, the ELISA for the 5′-tagged IFN-β failed, suggesting that the 5′-end tag abolished binding of the antibody with the protein (Fig. 2D). Despite this limitation, this result demonstrated that the bioluminescent peptide tagging system provides a faster and more robust quantification of recombinant proteins compared to ELISA.

**TABLE 1:** Performance comparison of luminescent assay and ELISA

	ELISA				Luminescent assay			
	Leptin_tag	IL-22_tag	IFN-β_tag	Tag_IFN-β	Leptin_tag	IL-22_tag	IFN-β_tag	Tag_IFN-β
Linear range (pg/mL)	114.93–999.91	13.37–103.59	193.13–289.31	Not detected	0.16–64,550	0.16–43,953	0.5–32213.9	Not detected

### *In situ* protein detection

**Fig 3:**
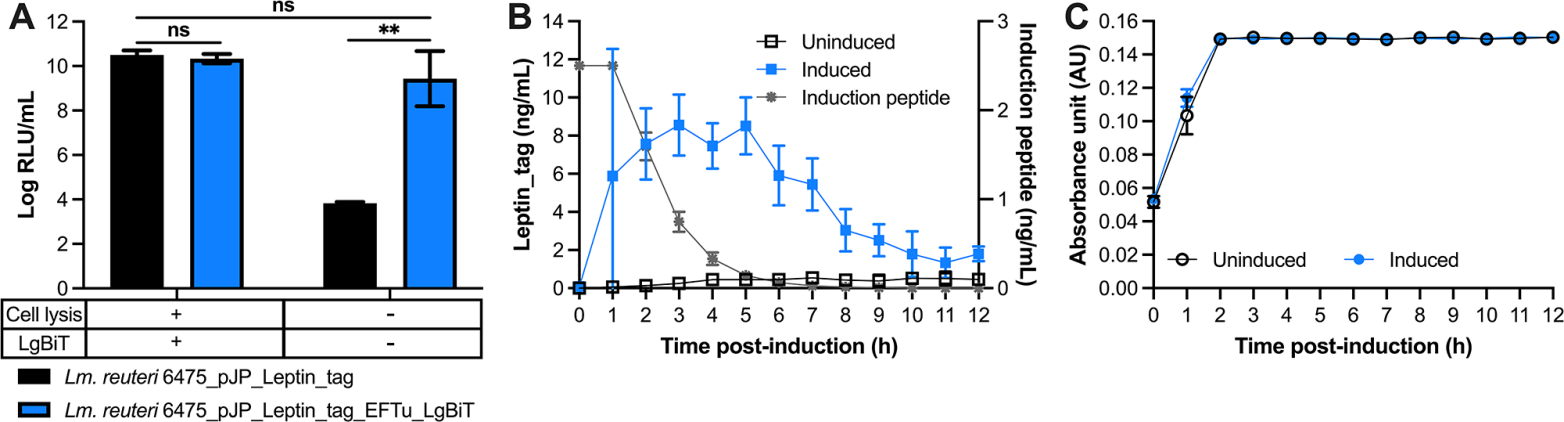
Real-time *in situ* detection of recombinant leptin in *Lm. reuteri*. (**A**) Luminescent signal of leptin_tag from *Lm. reuteri* harboring pJP_Leptin_tag and *Lm. reuteri* harboring pJP_Leptin_tag_EFTu_LgBiT with or without bacterial cell lysis and additional LgBiT. (**B**) Leptin and induction peptide concentrations during 12 h continuous culture of *Lm. reuteri* harboring pSIP_Leptin_tag_EFTu_LgBiT. (**C**) Maintenance of OD of *Lm. reuteri* harboring pSIP_Leptin_tag_LgBiT. Data are presented as means ± SD based on three biological replicates. **P < 0.01, ns; no significant differences (*P* > 0.05) (two-tailed paired *t*-test).

While the small luminescent tag can be used to identify and quantify recombinant proteins, bacterial lysis is required to release the recombinant protein. To overcome this bottleneck, we introduced the gene encoding LgBiT on a plasmid (see Fig. 1A) so an interaction can be formed between LgBiT and the luminescent tagged protein inside the cell. For *in situ* detection of recombinant proteins, it is required that the luminescent substrate enters the cell and that the luminescent signal from live cells can be detected. We determined that adding Nano-Glo HiBiT Extracellular Substrate yielded a luminescent signal in cells expressing the luminescent tagged protein and LgBiT, while no signal was obtained in cells lacking LgBiT (Fig. 3A). *In situ* protein levels were comparable to protein levels obtained following cell lysis (*P* > 0.05; Fig. 3A). Thus, protein production can be tracked in live bacterial cells.

We aimed next to determine *in situ* detection of recombinant protein production during continuous bioprocessing. We constructed *Lm. reuteri* harboring pSIP_Leptin_tag_EFTu_LgBiT where Leptin_tag is under the control of an inducible promoter and the LgBiT protein is expressed from the constitutive EFTu promoter. In a continuous bioreactor system, we cultured *Lm. reuteri* harboring pSIP_Leptin_tag_EFTu_LgBiT in two vessels each containing 200 mL of culture. During the early logarithmic phase [absorption units (AU) = 0.05, which is equivalent to OD_600_ = 0.3], one vessel was supplemented with induction peptide (2.5 ng/mL) to initiate the expression of leptin_tag. Samples were harvested every hour for up to 12 h (Fig. 3B). Once cells reached AU = 0.15 (OD_600_ = 1.0), approximately 2 h upon induction, the cell density was stably maintained at AU = 0.15 (Fig. 3C). Three hours following induction, maximum intracellular levels of leptin_tag were observed (8.9 ng/mL) after which leptin_tag levels gradually reduced (Fig. 3B). Modeling of the flow rate and induction peptide concentration over time revealed that 6 h post-induction less than 0.072 ng/mL of induction peptide would be present (Fig. 3B). Because of the continuous culture setup, the concentration of the induction peptide gradually reduced, which explains why we detected lower levels of leptin_tag over time. In the vessel without induction peptide, recombinant protein levels remained below 0.8 ng/mL. Collectively, these data revealed that the bioluminescent peptide tagging system can be applied for *in situ* detection of (recombinant) proteins and opens up exciting opportunities for optimization studies of protein production in microbial cell factories along with addressing fundamental questions on native protein production in different experimental setups.

### Tracking recombinant bacteria during gastrointestinal transit

**Fig 4:**
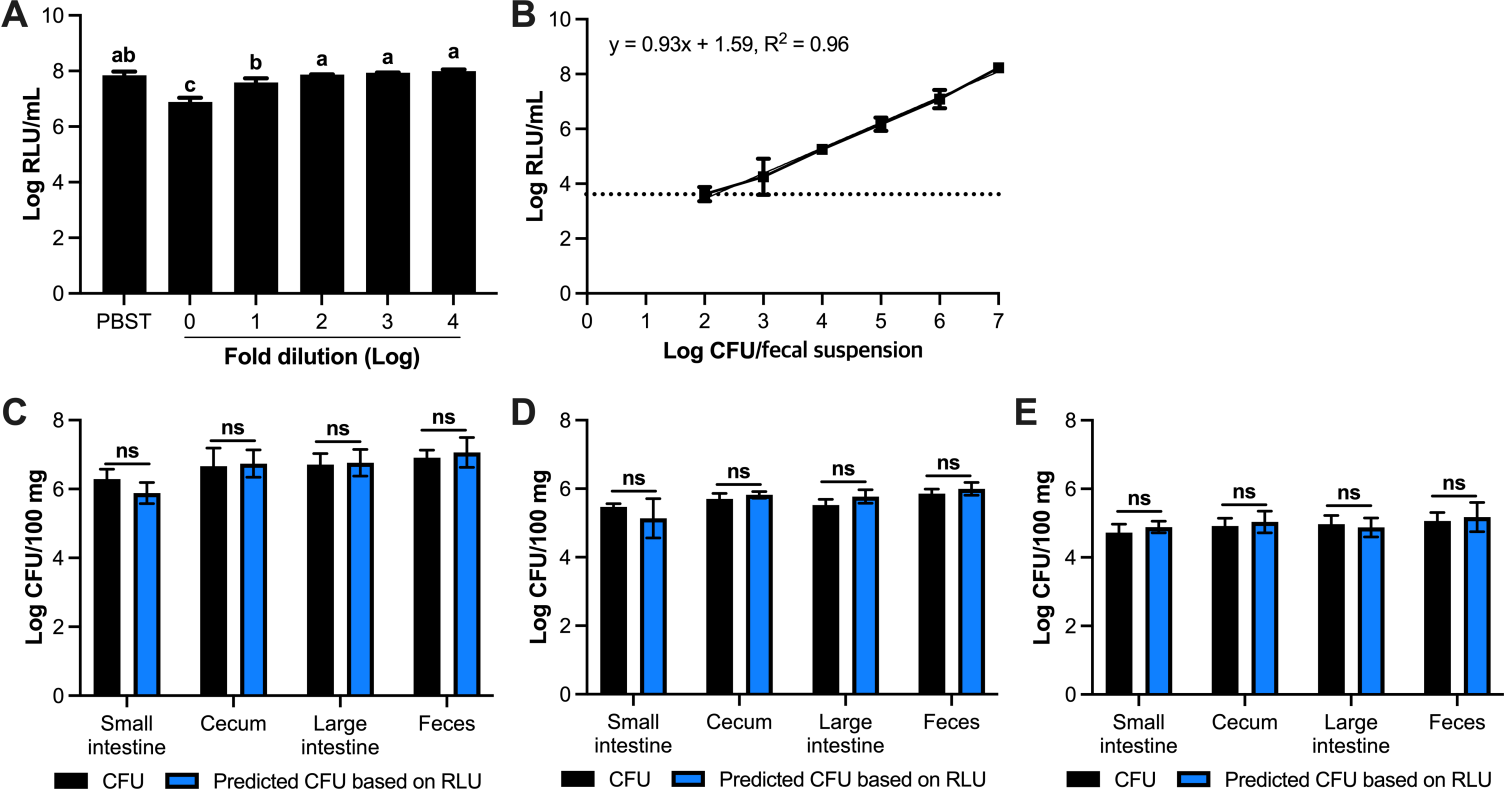
*In vivo* quantification by luminescence. (**A**) Diluting fecal material removes inhibitory factors impacting luminescent signal. (**B**) Standard curve of luminescent signal vs concentration of *Lm. reuteri* harboring pJP_Leptin_tag_EFTu_LgBiT in fecal suspension (1 mg/mL). (C–E) Detection of *Lm. reuteri* harboring pJP_Leptin_tag_EFTu_LgBiT throughout the mouse gut with different oral gavage dose at (**C**) 10^9^ CFU, (**D**) 10^8^ CFU, and (**E**) 10^7^ CFU. All data are presented as means ± SD based on three biological replicates. The letters (A –C) represent statistical differences between the groups (*P* < 0.05, one-way ANOVA). ns; no statistical significance (two-tailed paired *t*-test).

Now we have laid a foundation for the *in vitro* and *in situ* use of the bioluminescent peptide tagging system in *Lm. reuteri*, we next explored its applicability to quantify bacteria during and following gastrointestinal transit. First, we investigated to what extent mouse feces, a complex matrix, interferes with luminescence. *Lm. reuteri* harboring pJP_Leptin_tag_EFTu_LgBiT was inoculated in 1 mL of PBS-T, fecal suspension (100 mg/mL) in PBS-T, or in diluted fecal suspensions at the final concentration of 7-log CFU/mL. Compared to PBS-T, luminescence levels were 10-fold lower in the fecal suspension of 100 mg/mL (Fig. 4A). Diluting the fecal suspension 10-fold (or more) yielded comparable luminescence levels to those observed in PBS-T. Thus, analogous to what we observed in MRS medium, a simple dilution step removes the inhibitory signal.

Next, we constructed the dynamic range curve to predict the bacterial concentration based on relative light unit (RLU) (Fig. 4B). The luminescent signal was increased linearly according to increasing the concentration of *Lm. reuteri* harboring pJP_Leptin_tag_EFTu_LgBiT, as determined by the conventional plate count method, with a correlation coefficient of 0.96. In addition, we analyzed the sensitivity which referred to the change in signal along with a change in sample mass. Thus, the sensitivity was defined as the slope of the linear standard curve (23). The sensitivity and limit of detection were determined to be 0.928-log RLU/log CFU and 3-log CFU/100 mg of feces, respectively. Thus, in feces, luminescent *Lm. reuteri* can be detected over a broad linear range.

To investigate whether bacteria can be quantified by luminescence in different regions of the GIT, and in feces, we administered oral gavage mice with 10^7^, 10^8^, and 10^9^ CFU of *Lm. reuteri* harboring pJP_Leptin_tag_EFTu_LgBiT. Twenty-four hours following oral administration, fresh fecal pellets were collected and animals were subsequently sacrificed. We harvested contents from the large intestine, small intestine, and cecum. Using the conventional plate count method, we determined CFU levels. Luminescence levels of the various contents were determined in PBS-T. Using a standard curve we established based on fecal suspensions (Fig. 4B), we converted luminescence levels to CFU to predict the concentration of *Lm. reuteri* harboring pJP_Leptin_tag_EFTu_LgBiT, which we compared to the conventional plate counts (Fig. 4C through E). Our data revealed that the predicted concentration of *Lm. reuteri* harboring pJP_Leptin_tag_EFTu_LgBiT by luminescence was similar to the conventional plate count method (*P* > 0.05; Fig. 4C through E) even at the lowest doses of 7-log CFU. Thus, the bioluminescent peptide tagging system now opens up the exciting opportunity to quantify bacteria and *in situ* recombinant protein production throughout the GIT.

### Bioluminescent peptide tagging system is broadly applicable in LAB and *Bifidobacterium bifidum*

**Fig 5:**
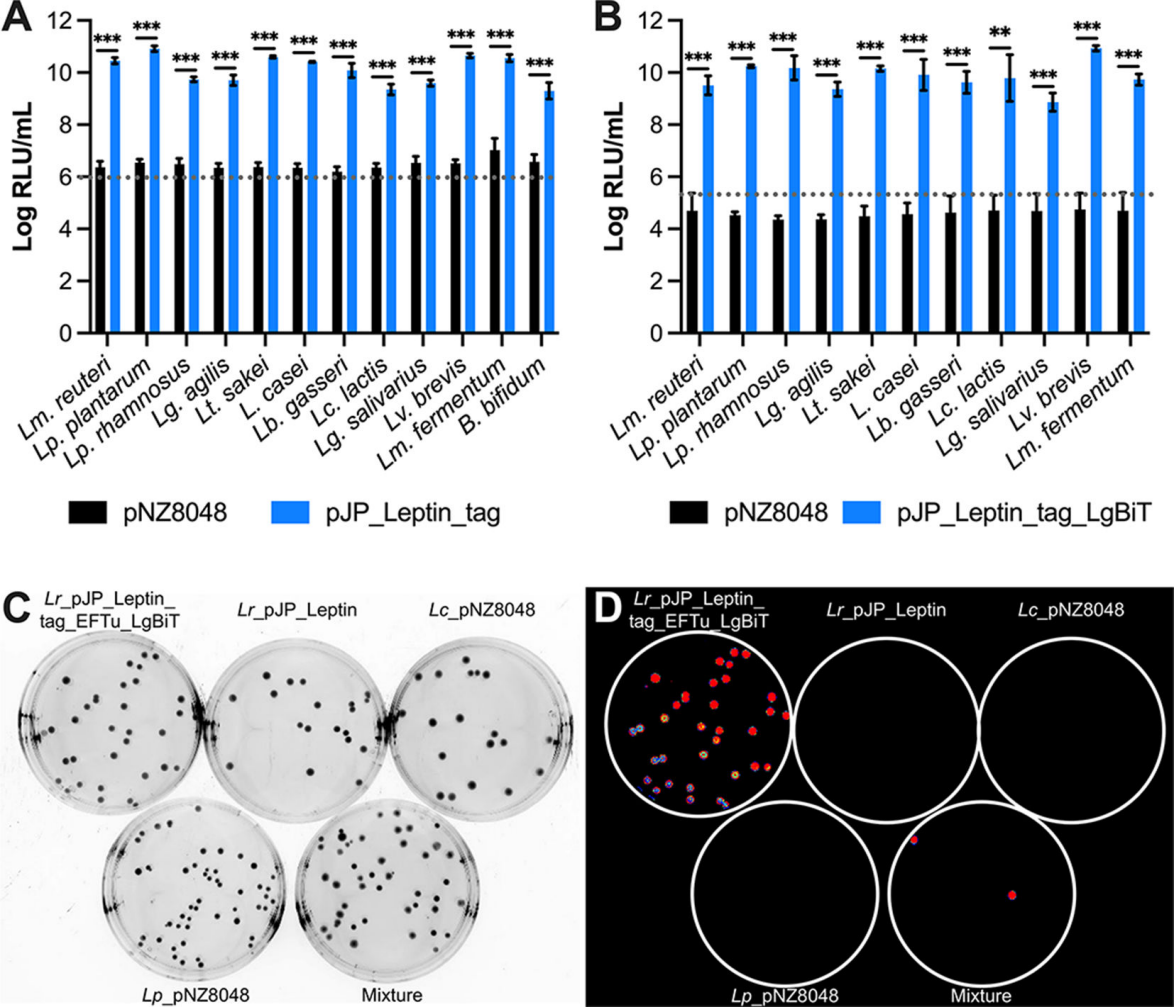
Bioluminescent peptide tagging is broadly applicable. (**A**) Luminescent signals in lysates derived from bacteria harboring pNZ8048 or pJP_Leptin_tag. (**B**) Luminescent signals from whole bacterial cells harboring pNZ8048 or pJP_Leptin_tag_EFTu_LgBiT. (**C**) Spread plates of four cultures including *Lm. reuteri* ATCC PTA 6475 (Lr)_pJP_Leptin_tag_EFTu_LgBiT, Lr_pJP_Leptin, *Lacicaseibacillus casei* BL23 VPL1021 (Lc)_pNZ8048, and *Lactiplantibacillus plantarum* ATCC BAA-793 (Lp)_pNZ8048, and a mixture thereof. (**D**) Luminescent imaging of colonies on plate. Data in panels A and B are presented as means ± SD based on three biological replicates. **P < 0.01, ***P < 0.001 (two-tailed paired *t*-test). Panels C and D are representative images from at least two independent experiments.

Now we have established the versatility of the bioluminescent peptide tagging system in *Lm. reuteri*, we next assessed the applicability of this bioluminescent system in other microbes. We established pJP_Leptin_tag or the control plasmid pNZ8048 in 11 different LAB strains (24) and in *Bifidobacterium bifidum*. Late log phase cultures were harvested by centrifugation, and cell pellets were disrupted by mechanical lysis. Following the addition of the luminescent substrate harboring LgBiT, luminescent signals were recorded (Fig. 5A). For all strains, luminescence levels were 3- to 4-log higher than luminescence levels obtained from lysates derived from cells harboring the pNZ8048 control plasmid.

Next, we assessed whether *in situ* measurement of recombinant protein is broadly applicable. To this end, bacteria were transformed with pJP_Leptin_tag_EFTu_LgBiT. Despite numerous attempts, we could not obtain transformants of pJP_Leptin_tag_EFTu_LgBiT in *B. bifidum*, which was therefore excluded from the analysis. Cultures were harvested at OD_600_ = 5, and cell pellets were mixed with the luminescent substrate that lacked LgBiT. Analogous to our findings with pJP_Leptin_tag_EFTu_LgBiT, all strains harboring pJP_Leptin_tag_EFTu_LgBiT yielded a significant increase in luminescence compared to the empty vector control pNZ8048 (Fig. 5B). Thus, the bioluminescent peptide tagging is broadly applicable among LAB, and we established proof of-concept in a *Bifidobacterium* spp.

Whole cell luminescence now creates opportunities to identify microbes from a mixed community, to identify genetic transformants, or to determine plasmid stability. As a proof of concept, we created five spread plates: (1) *Lm. reuteri* harboring pJP_Leptin_tag_EFTu_LgBiT; (2) *Lm. reuteri_*pJP_Leptin; (3) *L. casei*_pNZ8048; (4) *Lp. plantarum*_pNZ8048; and (5) a random mixture of cultures 1–4 (Fig. 5C). Plates were flooded with luminescent substrate and subjected to luminescent imaging (Fig. 5D). Colony luminescence was observed on all colonies present on the plate with *Lm. reuteri* harboring pJP_Leptin_tag_EFTu_LgBiT, whereas colonies derived from strains harboring the control plasmid pNZ8048 did not luminesce. Mixing different cultures easily led to the identification of *Lm. reuteri* harboring pJP_Leptin_tag_EFTu_LgBiT, which collectively demonstrates the proof of concept of the application of colony luminescence.

## DISCUSSION

LAB are extensively applied in the food industry for various fermentation processes, in medicine as probiotics, and in biotechnology as production factories of recombinant proteins (25). In this study, we developed a peptide-based bioluminescent tagging system and demonstrated its use in 11 different LAB encompassing eight genera, and *B. bifidum*. Although the 11 LAB strains belonged to a single genus previously, they were recently reclassified into eight different genera due to the high phenotypic, ecological, and genetic diversities (24). The successful implementation of the peptide-based luminescent system within such a diverse group of organisms means that our approach will be highly relevant to LAB of industrial interest and to generate live biotherapeutic products. In fact, we envision that the described applications to detect bacteria and their recombinant proteins *in vitro*, *in vivo*, and *in situ* will be a valuable asset to the microbiome community.

We determined that the substrate required for *in situ* luminescence is able to enter the cells, which opened up the possibility of *in situ* luminescence measurements during growth. We did not notice major fluctuations in luminescence levels between *in situ* measurements and lysates. This not only suggests that the uptake of the substrate appears efficient but also points toward the broad applicability of bioluminescent peptide tagging for *in situ*, at least in LAB. However, a current drawback is that this system described in this work is currently only applicable for short-term true real-time detection of protein production, mainly because of the relatively short half-life—less than 2 h—of the substrate in MRS medium. Nevertheless, the *in situ* quantification following sampling can provide useful insight into microbial physiology in response to different stimuli.

The luminescent peptide system also presents an opportunity to serve as a selection marker to identify crossover recombinants, particularly in members that belong to the *Lb. delbrueckii*, *Holzapfelia floricola,* and *Amylolactobacillus amylophilus* (24, 26). The vancomycin-sensitive phenotype in these strains means that the broadly applicable vancomycin-based counterselection system cannot be applied (27). Instead, the bioluminescent peptide tagging could be applied to identify the cells in which single-crossover homologous recombination has occurred or to identify the cells that have lost the suicide vector following a second homologous recombination event. This application would be straightforward since adding the bioluminescence substrate to an agar plate suffices to yield a luminescent signal (Fig. 5C and D). If a higher-throughput application would be required one could consider flow cytometry, analogous to what previously has been described by Santoro *et al*., who applied fluorescence-activated cell sorting (FACS) to identify Cre recombinase mutants based on GFP expression level (28). Likely, the luminescent detection will be favorable over this technique because fluorescence-based assays have lower sensitivity and lower signal-to-noise ratio due to autofluorescence (14). In addition to serving as a selection marker, we envision that the HiBiT tagging system can also be used to detect bacterial cell–cell interactions. By surface exposure of HiBiT in one microbe and LgBiT in another microbe, it is expected that the interaction of both microbes, for example within a biofilm, will result in a luminescent signal.

However, whatever the application, when developing a bioluminescent peptide tagging system in your microbe of interest, one may consider generating independent clones encoding a tag on either the N- or the C-terminal end. While it has been suggested that smaller tag sizes are less likely to disrupt protein folding or function (16), we observed that the presence of the 11-amino acid tag on the N-terminal end of IFN-β abolished detection by ELISA. This could be due to the tag affecting protein folding, thereby blocking the binding of the antibody, or it is also possible that the location of the tag influences the luminescence level for the analogous reason that the LgBiT protein may bind less efficiently. Regardless, once the location of the tag is optimized, the bioluminescence-based protein detection system will offer plenty of applications, including the quantification of engineered microbes in the mouse GIT. We also detected luminescent microbes throughout the mouse GIT following oral administration of 10^7^ CFU’s, a dose that is 100-fold lower compared to that used in most animal studies (29 – 32). This opens the possibility to perform higher-throughput time course studies on intestinal persistence, for example, where luminescence levels can be used to quantify microbes in feces in minutes, which we determined is comparable to the standard plate counts.

We also tested whether we could detect released recombinant proteins in the various regions of the GIT. Specifically, we engineered *Lm. reuteri* to accumulate recombinant leptin inside the cell, which is released during gastrointestinal transit via the activation of prophages (2, 33). Prophage-mediated release of recombinant proteins has proven fruitful in various preclinical models (7, 34). However, upon oral administration of the engineered *Lm. reuteri*, we could not detect an increase in luminescence in samples derived from the small intestine (data not shown). Attempts to detect luminescence following a boost in recombinant protein release by *in vitro* mitomycin-C induction, to induce prophages and thus increase therapeutic release, were unsuccessful (data not shown). Possibly, the amount of recombinant protein released was not enough to overcome the inhibitory effects of bile acids and digestive enzymes present in the small intestine (35), perhaps combined with the possibility of absorption of the recombinant protein by the intestinal epithelium (36). We are looking forward to revisiting the opportunity to track recombinant proteins in the GIT with constructs optimized to release recombinant proteins.

In conclusion, the bioluminescent peptide tagging system serves as a potent tool to monitor *in vitr*o and *in situ* protein production in a wide range of microbes, as well as for understanding the fate of probiotics in the GIT, their intestinal behavior, and ultimate localization. Its broad applicability opens the door to higher-throughput screening of probiotics and the development of next-generation probiotics for use in agriculture and in the clinic.

## MATERIALS AND METHODS

### Bacterial strains and growth conditions

The bacterial strains and plasmids used in this study are listed in Table 2. All lactobacilli strains and *Bifidobacterium bifidum* were cultured at 37°C in MRS medium (MRS, BD BioSciences) under hypoxic conditions (5% CO_2_ and 2% O_2_), and *Lactococcus lactis* strains were cultured in M17 (BD) containing 0.5% (w/v) of glucose at 30°C (static). *Escherichia coli* was grown at 37°C with gentle agitation (200 rpm) in lysogeny broth (LB, Neogen). If applicable, erythromycin was used at the concentrations of 300 µg/mL for *E. coli* and at 5 µg/mL for other strains listed in Table 2.

**TABLE 2:** Bacterial strains and plasmids used in this study^*a*^

Bacterial strains or plasmids	Characteristics	Source/Reference
*Escherichia coli* EC1000	in *trans* RepA provider, Kan^R^ (*E. coli* cloning host)	(37)
VPL3789	EC1000 harboring pVPL3789, Em^R^	(2)
VPL31599	Harboring pVPL31599, Em^R^	This study
VPL31655	Harboring pVPL31655, Em^R^	This study
VPL31948	Harboring pVPL31948, Em^R^	This study
VPL31964	Harboring VPL31964, Em^R^	This study
VPL31974	Harboring pVPL31974, Em^R^	This study
VPL31970	Harboring pVPL31970, Em^R^	This study
VPL31954	Harboring pVPL31954, Em^R^	This study
VPL31994	Harboring pVPL31994, Em^R^	This study
VPL31998	Harboring pVPL31998, Em^R^	This study
*Bifidobacterium bifidum* NRRL B-41410	Feces of breast-fed human infant	NRRL
VPL31866	Harboring pVPL31599, Em^R^	This study
VPL31885	Harboring pNZ8048, Em^R^	This study
*Ligilactobacillus agilis* VPL1214	Cow	Laboratory stock
VPL31875	Harboring pNZ8048, Em^R^	This study
VPL31854	Harboring pVPL31599, Em^R^	This study
VPL31936	Harboring pVPL31655, Em^R^	This study
*Levilactobacillus brevis* ATCC 8287	Green fermenting Sevillano variety olives	ATCC
VPL31887	Harboring pNZ8048, Em^R^	This study
VPL31868	Harboring pVPL31599, Em^R^	This study
VPL31950	Harboring pVPL31655, Em^R^	This study
*Laticaseibacillus casei* BL23 VPL1021	Cheese	(38), Cheese
VPL31879	Harboring pNZ8048, Em^R^	This study
VPL31858	Harboring pVPL31599, Em^R^	This study
VPL31940	Harboring pVPL31655, Em^R^	This study
*Limosilactobacillus fermentum* ATCC 14931	Fermented beets	ATCC
VPL31889	Harboring pNZ8048, Em^R^	This study
VPL31869	Harboring pVPL31599, Em^R^	This study
VPL31952	Harboring pVPL31655, Em^R^	This study
*Lactobacillus gasseri* ATCC 33323	-	ATCC
VPL31881	Harboring pNZ8048, Em^R^	This study
VPL31860	Harboring pVPL31599, Em^R^	This study
VPL31942	Harboring pVPL31655, Em^R^	This study
*Lactiplantibacillus plantarum* ATCC BAA-793	Saliva	ATCC
VPL31871	Harboring pNZ8048, Em^R^	This study
VPL31850	Harboring pVPL31599, Em^R^	This study
VPL31932	Harboring pVPL31655, Em^R^	This study
*Limosilactobacillus reuteri* ATCC PTA6475	This strain is also known as MM4-1A	BioGaia AB
VPL 3791	Harboring pVPL3789, Em^R^	(2)
VPL2048	Harboring pNZ8048, Em^R^	Laboratory stock
VPL4069	Harboring il-22 gene in Lm. reuteri chromosome	Laboratory stock
VPL31250	Harboring IFN-β	Laboratory stock
VPL31611	Harboring pVPL31599, Em^R^	This study
VPL31657	Harboring pVPL31655, Em^R^	This study
VPL31976	Harboring pVPL31974, Em^R^	This study
VPL31972	Harboring pVPL31970, Em^R^	This study
VPL31956	Harboring pVPL31954, Em^R^	This study
VPL32004	Harboring pVPL31998, Em^R^	This study
*Lactiplantibacillus rhamnosus* GG ATCC53103	Human fecal sample	ATCC
VPL31873	Harboring pNZ8048, Em^R^	This study
VPL31852	Harboring pVPL31599, Em^R^	This study
VPL31934	Harboring pVPL31655, Em^R^	This study
*Latilactobacillus sakei* ATCC 15521	Moto, starter of sake	ATCC
VPL31877	Harboring pNZ8048, Em^R^	This study
VPL31856	Harboring pVPL31599, Em^R^	This study
VPL31938	Harboring pVPL31655, Em^R^	This study
*Ligilactobacillus salivarius* VPL1245	Cow	Laboratory stock
VPL31883	Harboring pNZ8048, Em^R^	This study
VPL31864	Harboring pVPL31599, Em^R^	This study
VPL31946	Harboring pVPL31655, Em^R^	This study
*Lactococcus lactis* subsp. *cremoris* MG1363	Dairy starter	(39)
VPL2042	Harboring pNZ8048, Em^R^	Laboratory stock
VPL31862	Harboring pVPL31599, Em^R^	This study
VPL31944	Harboring pVPL31655, Em^R^	This study
Plasmid		
pVPL2042	pNZ8048 derivative; Cm^R^ was replaced by EM^R^, Em^R^	Laboratory stock
pVPL3789	pJP028_EFTu_noSP_Leptin, Em^R^	
pVPL31250	pJP025_ EFTu _murine_pmut_ThyA	Laboratory stock
pVPL31599	pJP028_ EFTu _noSP_Leptin_tag, Em^R^	This study
pVPL31655	pJP028_ EFTu _noSP_Leptin_tag_EFTu_LgBiT, Em^R^	This study
pVPL31948	pSIP411_Leptin_tag, Em^R^	This study
pVPL31974	pJP028_ EFTu _noSP_ IFN-β _tag, Em^R^	This study
pVPL31970	pJP028_ EFTu _noSP_IL22_tag, Em^R^	This study
pVPL31954	pSIP411_Leptin_tag_EFTu _LgBiT, Em^R^	This study
pVPL31994	pJP028_EFTu_noSP_ IFN-β, Em^R^	This study
pVPL31998	pJP028_EFTu_noSP tag_IFN-β, Em^R^	This study

^*a*^ VPL: Van Pijkeren Lab strain identification number; pVPL: Van Pijkeren Lab plasmid identification number; ATCC: American Type Culture Collection; LRRL: Agricultural Research Service Culture Collection; Kan^R^: kanamycin resistant; Em^R^: erythromycin resistant; Cm^R^: chloramphenicol resistant.

### Reagents and enzymes

Reagents were obtained from Thermo Fisher unless stated otherwise. To fuse DNA fragments, we used ligase cycle reaction (LCR) (40). For cloning and screening purposes, we used Phusion Hot Start Polymerase II and Taq polymerase (Denville Scientific), respectively. DpnI was used to digest methylated template DNA after backbone amplification. The DNA was phosphorylated with T4 polynucleotide kinase and ligated with T4 DNA ligase. Oligonucleotides and synthetic double-stranded DNA fragments were synthesized by Integrated DNA Technologies (IDT) and are listed in Table 3.

**TABLE 3:** Oligonucleotides used in this study

Oligonucleotides^*a*^	Sequence (5’ – 3’)	Description^*b*^
oVPL329	attccttggacttcatttactgggtttaac	Rev for screening pJP_Leptin_tag
oVPL659	tgccccgttagttgaagaag	Fwd for pSIP411
oVPL660	attctgctcccgcccttatg	Rev for pSIP411
oVPL1221	gcttgaaacgttcaattgaaatggca	Screening oligonucleotide for pJP_Leptin_tag
oVPL1326	ccagttggtaacaatgccatgt	Sequencing oligonucleotide for pSIP_Leptin_tag_EFTu_LgBiT
oVPL1366	taaagcaattactgatattgctg	Fwd for EFTu amplification
oVPL1447	cgaattaatagaaaaacattagtcaaatac	Fwd for pJP backbone
oVPL1448	taatgaaaacctcctgataatttacaag	Rev for pJP backbone
oVPL2115	aacacaagcattacgtaaactca	Rev for IL-22 amplification
oVPL2238	atggttccaattcaaaaagttcaagatg	Sequencing oligonucleotide for pJP_Leptin_tag
oVPL2239	atgttaccagttaatactcgttgtaaat	Fwd for IL-22 amplification
oVPL2351	taatctcgctttgattgttctatcg	Sequencing oligonucleotide for pJP_Leptin_tag
oVPL4252	tgatctttgaaccaaaattagaaaacc	Fwd for pJP backbone
oVPL4254	tatttaaaaaaattagttgatctttgaaccaaaattagaaaacc	Fwd starting at stop codon of leptin with 17 bases of tag sequence
oVPL4255	aacgccaaccactaacacattctggactaacatctaattgttg	Rev upstream of stop codon leptin with 16 bases of tag sequence
oVPL4307	gctttcgatagaacaatcaaagcgagattattaagagttgatagtaacacgaaataacat	Bridging oligonucleotide to fuse LgBiT and pJP backbone
oVPL4308	accaacgaaatcctcaagagtaaataccattaatgaaaacctcctgataatttacaagta	Bridging oligonucleotide to fuse LgBiT and EFTu
oVPL4309	gtatttgactaatgtttttctattaattcgtaaggaagataaatcccataagggcgggag	Bridging oligonucleotide to fuse pJP backbone and EFTu
oVPL4310	taaggaagataaatcccataaggg	Fwd for pJP backbone
oVPL4312	tcatcaagtgttatatagcggtc	Rev for LgBiT screening
oVPL4336	cattgagaagattgccgaaa	Fwd for LgBiT
oVPL4337	atttgatccgctgacaatcc	Sequencing oligonucleotide for LgBiT and EFTu
oVPL4339	cccgtctaaggaattgtcagat	Sequencing oligonucleotide for pSIP_Leptin_tag_EFTu_LgBiT
oVPL4418	gttagtggttggcgtttatttaa	Fwd for pJP_Leptin_tag backbone
oVPL4493	atgattaattataaacaattacaattac	Fwd for IFN-β amplification
oVPL4493	atgattaattataaacaattacaattac	Screening oligonucleotide for IFN-β
oVPL4494	attttgaaaattacgagttaaacgacg	Rev for IFN-β amplification
oVPL4538	caaccatatatgcaagaagt	Sequencing oligonucleotide for IL-22
oVPL4538	caaccatatatgcaagaagt	Screening and sequencing oligonucleotide for IL-22
oVPL4539	cgtgatttattacatttattagc	Sequencing oligonucleotide for Leptin
oVPL4540	ttggcgtgttcaacgttatt	Sequencing oligonucleotide for IFN-β
oVPL4540	ttggcgtgttcaacgttatt	Sequencing oligonucleotide for oligo for IFN-β
oVPL4543	ttgattacctccttatcatca	Rev for pSIP_Leptin_tag
oVPL4544	tctagactcgaggaattcggt	Fwd for pSIP_Leptin_tag
oVPL4848	aaacgccaaccactaaccattaatgaaaacctcctgataatt	Fwd starting at stop codon of IFN-β with 17 bases of tag sequence
oVPL4849	atttaaaaaaattagtattaattataaacaattacaattacaa	Rev upstream of stop codon IFN-β with 16 bases of tag sequence
**Recombinant DNA**		
gVPL71	atggtatttactcttgaggatttcgttggtgactgggaacagactgccgctta taatttagatcaagtattagaacaaggcggcgtgtcttcattgctccagaac ttggcagtttcagttactcctattcaacggattgttcggagtggggaaaatgc gctgaagattgatattcatgttattattccgtacgaaggattgtcagcggatc aaatggcacaaatagaagaagtttttaaagtggtatatccggtcgatgatc atcatttcaaagttattttaccttacgggaccttagtaattgatggtgtgactcc taatatgttaaactattttggacgtccttatgaaggaattgctgtttttgatggaa agaagattacagttacaggaactttgtggaatggaaataagattatcgatg agagattaattactccagatggaagtatgttatttcgtgttactatcaactcttaa	Codon optimized LgBiT

^a^ oVPL, van Pijkeren Laboratory primer identification number.

^b^ Fwd, forward oligonucleotide; Rev, reverse oligonucleotide.

### Construction of *Lm*. *reuteri*_pJP_Leptin_tag

To develop *Lm. reuteri* that produces HiBiT-tagged leptin, we first amplified the backbone of pVPL3789—a derivative of pNZ8048 that encodes murine leptin under the control of the EFTu promoter (2)—with oligo pair oVPL4254 and oVPL4255. Each oligonucleotide contained a 5′-end clamp that upon self-ligation yielded a 33-base tag that encodes HiBiT. Following DpnI treatment, phosphorylation, and self-circularization with T4 DNA ligase, the ligation reaction was introduced into *E. coli* EC 1000 by electroporation. By colony PCR (oVPL1447-oVPL329), putative clones were identified. The integrity of the purified plasmid DNA was determined by Sanger sequencing (Genewiz), and the resultant plasmid was named pJP_Leptin_tag (pVPL31599). Next, we transformed by electroporation pVPL31599 into *Lm. reuteri* VPL1014, resulting in *Lm. reuteri* harboring pJP_Leptin_tag (VPL31611). The integrity of the transformant was confirmed by amplification of the pJP_Leptin_tag backbone (oVPL2238-oVPL2351) followed by Sanger sequence analyses (oVPL2351).

### Proof-of-concept luminescence in *Lm. reuteri*

To release the intracellularly accumulated (tagged) leptin from bacterial cells, *Lm. reuteri* harboring pJP_Leptin (VPL3791) and pJP_Leptin_tag were diluted to OD_600_ = 0.1 and harvested after 8-h growth. After measuring the OD, cells were washed once with PBS-T (PBS with 0.05% (v/v) Tween 20) and resuspended in PBS-T. Subsequently, cells in MRS culture or PBS-T were disrupted by bead-beating. Briefly, approximately 200 µL of zirconia glass beads (BioSpecP) was added to 1 mL of cell suspension followed by two cycles of 1.5 min bead-beating with a 30-s interval on ice. Cell-free extracts were prepared by centrifugation (1 min, 9,391× *g*, 4°C) and filtered by a 0.2-µm-pore syringe filter (Argos Technologies). The luminescent signals were determined in a Glomax Discover Microplate Reader (Promega) using the HiBiT extracellular detection kit (Promega Co., Madison, WI, USA), following the manufacturer’s instructions. RLUs were normalized as RLU/the initial RLU ×5 (OD when the bacterial concentration was 9-log CFU/mL).

### Optimization of luminescence detection in *Lm. reuteri*

To investigate the inhibitory effect of pH on luminescent signal, cells derived from 1 mL of late log phase (OD_600_ = 4.5) *Lm. reuteri* harboring pJP_Leptin_tag were washed, as described above, and resuspended to 9-log CFU/mL in the original supernatant (non-pH adjusted; pH 4.6) or in the supernatant that was adjusted to pH 6.5 with 5 N NaOH. Subsequently, we prepared lysates and determined luminescence levels as described above.

To determine the extent to which MRS inhibits luminescence, we measured the luminescence of leptin_tag in different concentrations of MRS. First, we diluted MRS to 2-, 5-, 10-, 100-, and 1,000-fold with PBS-T (PBS with 0.05% (v/v) Tween 20). Late log phase of *Lm. reuteri* harboring pJP_Leptin_tag was prepared as shown above and resuspended into 1 mL (diluted) MRS to reach 9-log CFU/mL. Subsequently, we prepared lysates and performed luminescent signal analysis as described above.

To visualize bioluminescence in bacterial cultures, stationary phase cultures of *Lm. reuteri* harboring pJP_Leptin_tag (VPL31611) were centrifuged (1 min, 9,391× *g*, 4°C), and the cell pellets were washed once with PBS-T. Cell suspensions were used to prepare the lysate as described above. The lysate was twofold serially diluted into PBS-T and 100 µL of each diluted sample was added in a 96-well plate. The luminescent signal of each sample was measured using the HiBiT extracellular detection kit as described above with the Glomax Discover Microplate Reader. The bioluminescence image of the same 96-well plate was obtained with the ChemiDoc imaging system (Bio-Rad), using the chemiluminescent filter. The luminescent intensity was visualized using the ImageLab software (Bio-Rad, version 6.1).

To investigate the dynamic range by which CFU can be quantified using luminescent analysis, we measured the luminescent signal of leptin_tag obtained from different concentrations of *Lm. reuteri* harboring pJP_Leptin_tag. Late log phase culture was harvested, washed, and resuspended into PBS-T or MRS to reach the final concentration of 9-log CFU/mL. Each bacterial suspension was 10-fold serially diluted into PBS-T. After dilution, cells underwent the same procedure to prepare samples for luminescent analysis.

### Comparison of bioluminescent-based quantification and commercial ELISA

To compare luminescent-based quantification of the HiBiT tag with commercial ELISAs, we constructed two additional plasmid constructs including pJP_IL-22_tag (pVPL31970) and pJP_IFN-β_tag (pVPL31974). First, we amplified a backbone from pJP_Leptin_HiBiT with oligonucleotide pair oVPL1448-oVPL4418 to generate a plasmid backbone lacking the leptin gene. *Il-22* and *ifn-β* genes were amplified with oVPL2239-oVPL2115 and oVPL4493-oVPL4494 from *Lm. reuteri* harboring pHelp_IL22 (VPL4069) or pJP_IFN-β_pMut_*thyA* (pVPL31250), respectively. Each gene was cloned into the pJP_Leptin_HiBiT backbone by LCR, as described previously (40) with bridging oligonucleotides oVPL4688-oVPL4490 for *il-22* and oVPL4689-oVPL4496 for *ifn-β*. Each LCR product, including pJP_IL-22_tag and pJP_IFN-β_tag, was transformed into *E. coli* EC1000. The insertion of *il-22* and *ifn-β* genes was confirmed by mismatch amplification mutation assay PCR (41) with oligonucleotide oVPL1447-oVPL4535-oVPL329 and oVPL1447-oVPL4493-oVPL329, respectively. The insertion of *il-22* and *ifn-β* genes was analyzed by Sanger sequencing with oVPL2115-oVPL2351 and oVPL1447-oVPL2351-oVPL4494, respectively. Finally, each plasmid was transformed to *Lm. reuteri* to generate *Lm. reuteri* harboring pJP_IL-22_tag and *Lm. reuteri* harboring pJP_IFN-β_tag. To add the sequence encoding the HiBiT tag to the 5′-end of the *ifn*-β gene, the backbone was amplified from pVPL31974 with the oVPL4494-4252, followed by blunt-end ligation and transformation. The deletion of the tag gene was analyzed by Sanger sequencing with oVPL1447-oVPL2351. Next, we constructed pJP_tag_IFN-β (pVPL31998) using pJP_IFN-β (pVPL31994) with oVPL4848-oVPL4849 which contains the HiBiT gene at the 5′-end following the method for pJP_Leptin_tag as described above. After transformation into *E. coli*, the Sanger sequencing was performed with oVPL1447-oVPL2351 to identify the insertion of HiBiT.

Stationary phase *Lm. reuteri* strains harboring pJP_Leptin_tag, pJP_IL-22_tag, or pJP_IFN-β_tag were inoculated separately into MRS + EM5 to OD_600_ = 0.1. Once the cell density reached OD_600_ 4–5, cells were washed and disrupted in PBS-T as stated above. The lysates were serially diluted in ELISA dilution buffer or PBS-T and quantified by ELISA (R&D Systems Inc., Minneapolis, MN, USA) and HiBiT extracellular detection kit.

### *In situ* detection of recombinant proteins

To detect recombinant proteins *in situ*, we engineered *Lm. reuteri* to produce the LgBiT protein that interacts with HiBiT to yield a luminescent signal. Briefly, the LgBiT sequence was codon optimized for expression in *L reuteri* using the OPTIMIZER web server (42, 43) and synthesized by Integrated DNA Technologies (Table 3). The synthesized LgBiT product was named gVPL71. We amplified gVPL71 with oligonucleotide pair oVPL4326-oVPL4327, and the EFTu promoter was amplified from pJP_Leptin (VPL3791) with oligonucleotide pairs of oVPL1447 and oVPL1448. All these amplicons were then subjected to the LCR to fuse the three amplicons with bridging oligonucleotides of oVPL4307, oVPL4308, and oVPL4309. The LCR product was transformed into *E. coli* EC1000, and the cloning of the EFTu-LgBiT fusion into pJP_Leptin_tag was confirmed by PCR using oVPL1221 and oVPL4312. The plasmid sequence was analyzed by Sanger sequencing with oVPL1221 and oVPL4337. The resultant plasmid was named pJP_Leptin_tag_EFTu_LgBiT (pVPL31655). Finally, we transformed pVPL31655 into *Lm. reuteri* VPL1014 to yield *Lm. reuteri* harboring pJP_Leptin_tag_EFTu_LgBiT (VPL31657).

To detect leptin *in situ*, stationary phase cultures of *Lm. reuteri* harboring pJP_Leptin_tag (VPL31611) and *Lm. reuteri* harboring pJP_Leptin_tag_EFTu_LgBiT (VPL31657) were diluted to OD_600_ = 0.1 followed by culture until late log (OD_600_ = 4.5). Each culture was subsequently divided into two groups of which one of the cultures was subjected to bead-beating. The luminescent signal of each sample was measured using the HiBiT extracellular detection kit with or without LgBiT.

### *In situ* detection of recombinant protein during the continuous bioprocessing

To investigate the applicability of a bioluminescent peptide tagging system for *in situ* detection of recombinant protein, we cloned the gene encoding murine leptin with at the 3′-end the sequence encoding HiBiT downstream of the inducible promoter of plasmid pSIP411; downstream we cloned the EFTu promoter fused to the sequence encoding LgBiT to yield pVPL31952. Briefly, the backbone was amplified from pSIP_Leptin_HiBiT (pVPL31948) with oligonucleotide pairs of oVPL4543 and oVPL4544. The fusion of EFTu-LgBiT was amplified from pJP_Leptin_tag_EFTu_LgBiT (pVPL31657) using oVPL1366 and oVPL1221. The amplicons were mixed at a 1:1 molar ratio for blunt-end ligation, followed by electroporation into *E. coli* EC1000. The insertion of leptin_tag and EFTu_LgBiT was confirmed with the oligonucleotides oVPL659-oVPL4539-oVPL660 and oVPL659-oVPL4337-oVPL660, respectively. The integrity of the DNA sequence was confirmed by Sanger sequencing using oligonucleotides oVPL4339, oVPL4539, oVPL1326, and oVPL1447.

We used the DASbox Mini Bioreactor System (Eppendorf, 76D × 04 MB) to continuously culture *Lm. reuteri* harboring pSIP_Leptin_tag. We used two continuously operated stirred bioreactor vessels in parallel. Each bioreactor vessel contained 200 mL of MRS supplemented with 5 µg/mL of erythromycin and was operated with 2% of dissolved oxygen with agitation (50 rpm) at 37°C. The pH was maintained within a range of 6.3–6.7 and was adjusted with 1.5 N NaOH (Sigma-Aldrich). A stationary culture of *Lm. reuteri* harboring pSIP_Leptin_tag_EFTu_LgBiT was inoculated in each vessel to AU = 0.011 (OD_600_ = 0.1). At an AU of 0.050 (OD_600_ = 0.3), induction peptide was added to a final concentration of 2.5 ng/mL. For the continuous bioprocessing, fresh MRS containing 5 µg/mL of erythromycin was added at a flow rate of 300 mL/h to sustain the AU of 0.15 (OD_600_ = 1.0). To track *in situ* recombinant protein production, samples were aseptically collected every hour and luminescence was measured instantly using the above-described procedures.

### Microbial luminescence in murine feces

To examine the inhibitory effect of murine feces on luminescence detection, we measured the luminescent signal from *Lm. reuteri* harboring pJP_Leptin_tag_EFTu_LgBiT in different fecal dilutions. The fecal suspension (100 mg/mL) was 10-fold serially diluted in PBS-T. Late log phase *Lm. reuteri* harboring pJP_Leptin_tag_EFTu_LgBiT (OD_600_ = 4.5, 9-log CFU/mL) was prepared as described above followed by inoculation in each diluted fecal suspension or PBS-T to reach a final concentration of 7-log CFU/mL. The luminescent signal was measured as described above.

The standard curve of luminescent signal vs bacterial concentration in the fecal sample was constructed by measuring the *in situ* luminescent signal from different concentrations of *Lm. reuteri* harboring pJP_Leptin_tag_EFTu_LgBiT. The late log phase bacteria were prepared in PBS-T as stated above and inoculated into 100-fold diluted fecal suspension (1 mg/mL) to reach a final concentration of 2- to 7-log CFU/1 mL of fecal suspension. After then, the luminescent signal was measured from each suspension. The standard curve for quantification of bacterial cells was constructed to represent the relationship between bacterial concentration and luminescent signal.

### Microbial luminescence during gastrointestinal transit

All mouse experiments were performed in accordance with NIH guidelines, Animal Welfare Act, and US federal law and were approved by the Application Review for Research Oversight at Wisconsin (ARROW) committee and overseen by the Institutional Animal Care and Use Committee (IACUC) under protocol ID A006078-R1. Conventional pathogen-free and germ-free mice were housed at the Animal Science and Laboratory of Animal Research Facilities, respectively, at the University of Wisconsin-Madison.

Eight-week-old male B6 mice (C57BL/6J) were purchased from Jackson Labs (Bar Harbor, ME) and adapted for 1 week to the new environment prior to the start of the experiment. Animals were housed at an environmentally controlled facility with a 12-h light and dark cycle. Standard chow diet (LabDiet 5008) and water were provided *ad libitum*.

For oral administration, bacteria were prepared as follows. *Lm. reuteri* harboring pJP_Leptin_tag_EFTu_LgBiT was cultured in fresh MRS until OD_600_ = 2. Cells were washed and resuspended in PBS to adjust the concentration at 8-, 9-, and 10-log CFU/mL. Mice were administered oral gavage bacteria (100 µL of each concentration of cell) or PBS (*n* = 5/group). At 24 h post-gavage, each mouse was sacrificed, and the contents from the large intestine, small intestine, cecum, and feces were harvested. Each sample was resuspended in PBS to 100 mg/mL, and the luminescent signal was measured from sample suspension as described above. To predict the bacterial concentration, the luminescent signal was subjected to a preconstructed standard curve between the luminescent signal and bacterial concentration in fecal samples. In addition, all samples were plated on MRS agar containing 5 µg/mL of erythromycin to compare the bacterial concentration determined by RLU.

### Bioluminescent peptide tagging in LAB and *Bifidobacterium bifidum*

To investigate to what extent the bioluminescent peptide tagging system can be applied in other bacteria, we established by electroporation pJP_Leptin_tag in 10 different LAB species and one strain of *B. bifidum*. The plasmid pJP_Leptin_tag_EFTu_LgBiT could only be established in the LAB strains (Table 2). Competent cells were prepared as described by Oh et al. (44). Successful transformants were confirmed in an identical manner as described for the method of the construction of *Lm. reuteri* harboring pJP_Leptin_tag and *Lm. reuteri* harboring pJP_Leptin_tag_EFTu_LgBiT. All strains harboring pJP_Leptin_tag and pJP_Leptin_tag_EFTu_LgBiT were used for luminescent analysis following bead-beating and *in situ* luminescent analysis, respectively, as described above.

### Bioluminescence image analysis of cultures and colonies

To identify bioluminescent bacteria on agar plates, we plated stationary phase cultures of *Lm. reuteri* harboring pJP_Leptin_tag (VPL31611), *Lm. reuteri* harboring pJP_Leptin_tag_EFTu_LgBiT (VPL31657), *L. casei*_pNZ8048 (VPL31879), *Lp. plantarum*_pNZ8048 (VPL31871), or the four-strain mixture, on MRS agar plates (5 mm diameter) supplemented with 5 µg/mL erythromycin. After incubation at 37°C for 48 h, 1 mL of Nano-Glo HiBiT Extracellular Buffer containing 10 µL of Nano-Glo HiBiT Extracellular Substrate was poured on the agar plate. The colonies were visualized by the ChemiDoc imaging system with colorimetric and chemiluminescent filters, and images were analyzed using the ImageLab software.

### Statistical analysis

A minimum of three biological replicates were performed, and the results were expressed as mean ± SD. All samples were included in the analyses, and experiments were performed without blinding. Graphs were prepared using GraphPad Prism software (GraphPad Software, San Diego, CA, USA). All statistical analyses were performed using paired *t*-test and one-way analysis of variance (ANOVA) (GraphPad Prism, version 9). For Pearson correlation, we performed multivariate pairwise correlations (JMP pro 11.0.0).

## SUPPLEMENTAL MATERIAL

Fig. S1 mbio.01197-23-s0001.docx Comparison of dynamic range between the HiBiT peptide tagging luminescent assay and ELISA.

ASM does not own the copyrights to Supplemental Material that may be linked to, or accessed through, an article. The authors have granted ASM a non-exclusive, world-wide license to publish the Supplemental Material files. Please contact the corresponding author directly for reuse.
