# Supplementary material for: Bioluminescent monitoring of recombinant lactic acid bacteria and their products
Source: mBio. 2023 Sep 5;14(5):e01197-23. doi: 10.1128/mbio.01197-23 (PMC10653940; doi:10.1128/mbio.01197-23)
Supplement: Fig. S1 — Comparison of dynamic range between the HiBiT peptide tagging luminescent assay and ELISA. [file mbio.01197-23-s0001.docx]

**Supplemental Material**

**Bioluminescent monitoring of recombinant lactic acid bacteria and their products**

**Running title:** Bioluminescent assay to track lactic acid bacteria

**In Young Choi, Jee-Hwan Oh, Zhiying Wang and Jan-Peter van Pijkeren^#^**

Department of Food Science, University of Wisconsin-Madison, Madison, WI, 53706, USA

^#^Correspondence:

Phone: +1 608 890 2640

Fax: +1 608 262 6872

Email: [vanpijkeren@wisc.edu](mailto:vanpijkeren@wisc.edu)

**
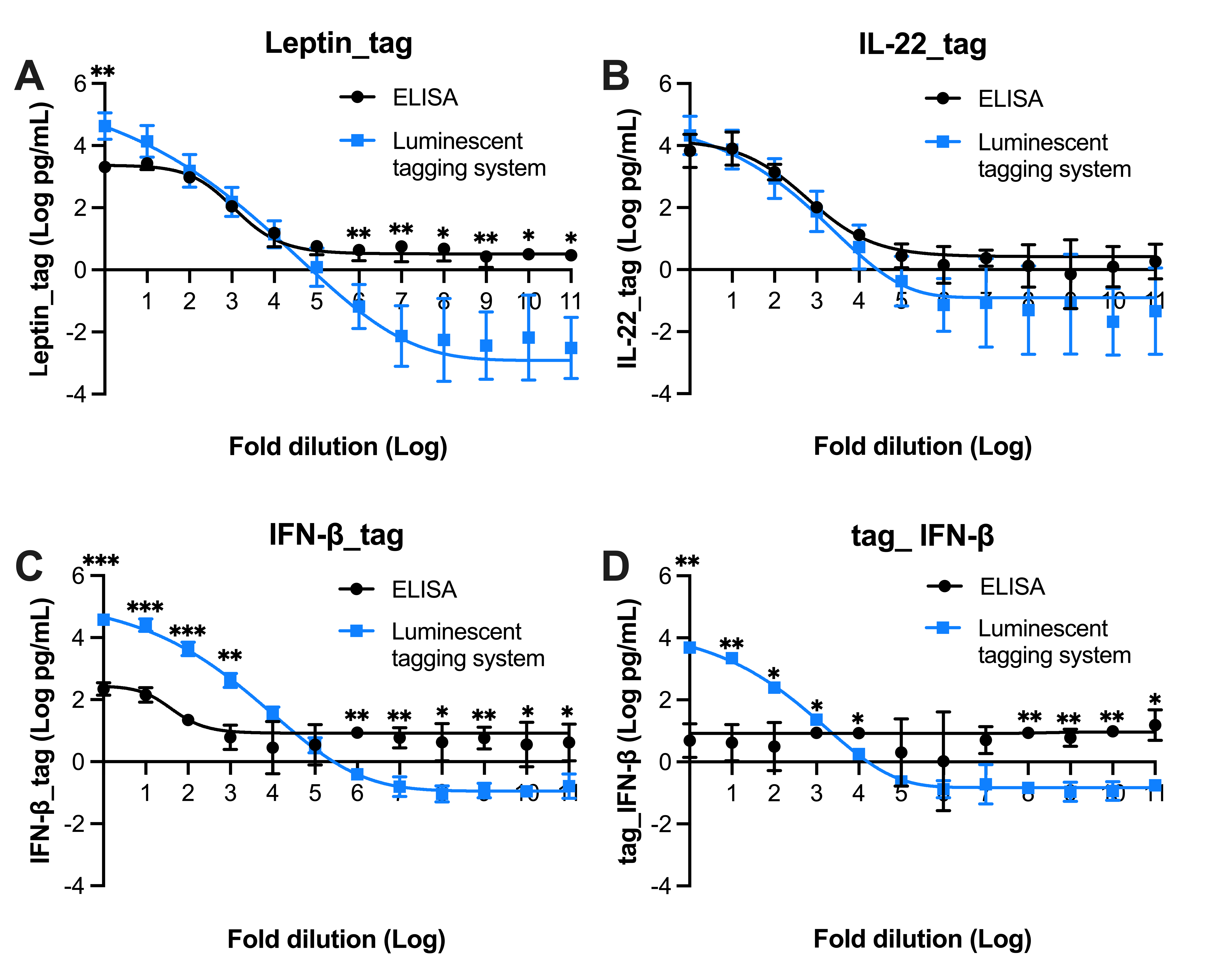
**

**Fig. S1.** Comparison of dynamic range between the HiBiT peptide tagging luminescent assay and ELISA. (A - D) dynamic range of protein concentration in Log pg/mL of (A) leptin_tag, (B) IL-22_tag, (C) IFN-β_tag, and (D) tag_ IFN-β as determined by ELISA (black lines) or bioluminescence (blue lines).

Data are presented as means ± S.D. and represent three biological replicates. *: p < 0.05; **: p < 0.01; ***: p < 0.001; ns; no significant differences between ELISA and bioluminescent tagging system (p > 0.05) (two-tailed paired t-test).
